# Seroprevalence and risk factors of hepatitis B and C virus infections in female workers of Lao garment factories

**DOI:** 10.1371/journal.pone.0199919

**Published:** 2018-07-16

**Authors:** Kinnaly Xaydalasouk, Michel Strobel, Yves Buisson, Antony P. Black, Claude P. Muller

**Affiliations:** 1 Institut de la Francophonie pour la Médecine Tropicale, Vientiane, Lao PDR; 2 Lao Lux Laboratory, Institut Pasteur du Laos, Vientiane, Lao PDR; 3 Department of Infection and Immunity, Luxembourg Institute of Health, Esch-sur-Alzette, Grand-Duchy of Luxembourg; Centers for Disease Control and Prevention, UNITED STATES

## Abstract

The prevalence of hepatitis B and C virus infections may be higher in vulnerable populations or in individuals likely to be exposed through risk behaviors such as female garment factory workers in Lao People’s Democratic Republic. A cross-sectional study was performed on 400 female garment workers in Vientiane Capital. Women were tested for hepatitis B virus surface antigen and antibodies against hepatitis B core, surface antigen and hepatitis C virus using commercial Enzyme-linked immuno-absorbent assays. Participants completed a standardized questionnaire about potential risk factors for both infections. Sixteen women (4±1.9%) were HBsAg carriers, 187 (47%) had anti-HBc, 116 (29%) anti-HBs and 7 (1.8±1.3%) anti-HCV antibodies. Three factors were significantly associated with the presence of anti-HBc (indicating previous exposure to HBV): (i) residence in dormitories, (ii) more than one sexual partner, (iii) history of abortion. Despite a high risk of exposure, the prevalence of anti HBV and anti HCV infection markers in this sample of female workers was not higher than in the Lao general population. Our data suggest that exposure to HBV happens later during life and was significantly associated with sexual risk behavior. Thus, this study highlights the vulnerability of these women who were mostly young, uneducated, unvaccinated, of rural origin and were not aware of the risk of infections. An occupational health program targeting the female factory workers should be implemented in Lao PDR.

## Introduction

Hepatitis B virus (HBV) is mainly transmitted by blood transfusions, unsafe injection practices, tattooing, occupational hazards, sexual risk behaviors, or mother-to-child in the perinatal period [1]. In Lao PDR, the seroprevalence of chronic HBV as indicated by the presence of surface antigen (HBsAg) has been estimated at 6–9% (blood donors and the general population), while the rate of exposure to HBV, as measured by the presence of anti-HBc antibodies, reaches 40% to 50% in the adult population [1][2][3][4].

Hepatitis C virus (HCV) is mainly acquired through blood exposure, via transfusions, use of unsafe injections in health care settings and by drug abusers. Sexual transmission and mother to child transmission are infrequent [5]. The prevalence of anti-HCV antibodies has been estimated at 1.1% among Lao blood donors [2].

In Southeast Asia, most chronic HBV carriers have been infected at birth or during infancy [1]. Later in life, infections with HBV as well as HCV infections may be indicative of the aforementioned risk behaviors. Young female factory workers in resource-limited countries such as Lao PDR could represent a particularly vulnerable population with an enhanced risk of exposure to these infections. Indeed, women from remote villages, with low level of education, may find themselves in difficult working and living conditions, away from the protective environment of their family and community, and at risk of human and sex trafficking [6]. Health data about this vulnerable group are limited. Risk factors for infectious diseases among factory workers have never been studied in Lao PDR. Furthermore working conditions in garment factories supplying international markets have raised international criticism.

For these reasons, we conducted a cross-sectional study to determine the prevalence of hepatitis B and C virus infections and associated risk factors in female workers employed in Lao garment factories.

## Materials and methods

### Study population

This cross-sectional study was conducted among female garment factory workers in Vientiane capital where a large number of such factories are located. The five largest garment factories were selected. Four hundred women were enrolled, corresponding to almost 10% of all female workers employed in the 5 factories (April to July 2015). This sample was well above the minimum required size of 315 women, based on a prevalence of HBV infection estimated at 8% with a precision of ±3% and an alpha risk of 5%. If using the same formula for anti-HCV with an estimated prevalence of 1.1% and precision of ±1.02, the sample size is 400.

In each factory, the participants were randomly selected from the Lao trade union list, proportionally to the total number of women employed. The questionnaire was designed according to the conceptual framework (S1 Fig) and included socio-demographics (age, income, education level, province of origin, family status), knowledge about HBV and HCV infections (in particular, routes of infection), risk behavior and practices of prevention.

The aim of the study was clearly explained by the research team to all participants. An information sheet stated that participation was voluntary and that consent could be withdrawn at any time. An oral explanation was given directly to illiterate employees. All participants signed a letter of consent. The illiterates gave their agreement in the presence of witnesses. Two workplaces were set up, one for interviews and another one for blood sampling. The confidential interviews were held in places assigned by the factory management, i.e. cafeteria (during off-hours), conference room, or first aid room.

All participants received information about sexually transmitted diseases in Lao language. HBsAg positive participants were contacted and invited to a Central Hospital or a clinic for Lao youth, where they were counselled about their HBV status and about further actions.

The study was conducted according to Good Clinical Practice (GCP) and international guidelines. Approval to conduct the study was obtained from the National Ethics Committee of Lao PDR (No.034/2015).

### Blood testing for viral hepatitis markers

A 5 ml sample of venous blood was collected from each participant into a dry tube, transported to the laboratory at 4°C and centrifuged to separate the serum which was stored at -80°C until testing. Commercial ELISA tests (Diasorin, Italy) were used to detect HBsAg, anti-HBs, anti- HBc and anti-HCV antibodies. HBsAg was only tested if anti-HBc were positive and anti-HBs negative. Previous studies in Lao PDR have shown that approximately 98% of the HBsAg positive individuals have this serological profile[7]. Thus, we may miss a small proportion of HBsAg positive participants for example those with early acute infection. HBsAg positivity was interpreted as a marker of chronic infection, although in some case it may be indicative of an acute HBV infection. Anti-HBc without HBsAg was indicative of a previous HBV infection and anti-HBs positivity without anti-HBc was interpreted as previous vaccination.

### Statistical analysis

A multivariate logistic regression model was used to identify factors associated with the presence of anti-HBc antibodies. This model was built based on bivariate analyses with a 20% significance level as threshold for inclusion in the multivariate model. Factors that were considered highly important were included in the multivariate even if the 20% significance level was not reached in the bivariate analysis.

## Results

### Population characteristics

Only six of the randomly selected women refused to participate for reasons such as fatigue, pregnancy, dizziness or fear of blood drawing. They were replaced with another 6 randomly selected participants. In total, 400 female workers from the five garment factories of Vientiane Capital agreed to participate in the survey and were included from April to July 2015. We were unable to draw blood from one of the participants. The median age was 25 (range 15–57 years) and their average income was 1,250,740 ± 591,842 Lao Kip (LAK) per month* (range 300,000–5,000,000 LAK) [1 US dollar equals approximately 8100 LAK]. Twelve women (3%) were illiterate, 147 (39.75%) had only primary school education and only 25 women (6.25%) had a level of education higher than high school. Most women (77.50%) came from provincial areas. Over half of the participants were unmarried, but 253 women (63.25%) had a boyfriend. Approximately half of women (51.25%) lived in dormitories of the factories, while 150 (37.5%) live in their house and 45 (11.25%) rented a room. Overall, 174 (43.5%) lived with family, 221 (55.3%) lived with friends, and 5 (1.3%) lived alone. Regarding their gynecological history, 145 (36.25%) had given birth and 91 (22.75%) had had an abortion.

### Knowledge, attitudes and practices

Nearly a third of women (31.5%) were aware of hepatitis but none had heard of HCV. Very few knew about the routes of transmission of HBV and HCV (2.75%), the symptoms or complications caused by these viruses (6.5%), including liver failure, cirrhosis and cancer. Only 5.25%, misunderstood that there is a cure for hepatitis B but less than 1.0% knew how to protect themselves from HBV infection. Importantly, only 6.25% were aware of the existence of vaccination against HBV.

Of all the women surveyed, 6.75% reportedly had previously been tested for HBV infection and only one claimed having been vaccinated against HBV. One worker recalled the stigma associated with HBV infection. Most women (96.5%) said they would inform their families if they learned they were infected with HBV or HCV. Few women (3%) said they would avoid any contact with a person infected with HBV if this person was a family member.

### Risk factors

Among factors of blood exposure, 13% of women reported a surgical history, 4% had received one or more blood transfusions and 26% mentioned behaviors or risk activities such as tattoos, piercings, injected drug use and reuse of needles.

About the risk of sexual transmission, out of 269 women who had at least once sexual relationship, 57 (21.2%) had more than one sexual partner and 146 (54.0%) did not use condoms.

Six of these workers (2.2%) had had sex for money and 27 (10%) reported a history of sexually transmitted infection (STI).

### Prevalence of hepatitis B and hepatitis C markers

Of 187 (46.9%) anti-HBc positive women, 84 (44.9%) were anti-HBs negative, of whom 16 were HBsAg carriers, i.e. 4±1.9% of the total sample. 7 participants (1.8±1.3%) were positive for anti-HCV, one of whom was also HBsAg positive. Only 3.3% had a profile indicating vaccination (anti-HBs positive, anti-HBc negative) (Table 1).

**Table 1:** Serological markers of hepatitis B and C.

Serological profile	n	%	95% CI	Interpretation
Anti-HBs negative/anti-HBc negative	200	50.13	45.0–55.0	No HBV infection, no vaccination
Anti-HBs positive/anti-HBc negative	13	3.26	2.0–5.0	HBV vaccination
Anti-HBs positive/anti-HBc positive	103	25.81	22.0–30.0	Previous HBV infection
Anti-HBs negative/anti-HBc positive/HBsAg negative	67	16.79	13.0–20.0	Previous HBV infection
Anti-HBs negative/anti-HBc positive/HBsAg positive	16	4.01	2.1–5.9	Acute or chronic HBV infection
Anti-HCV positive*	7	1.75	0.5–3.0	Current or previous HCV infection

*1 out of the 7 HCV positive participants had current co-infection with HBV

### Bivariate analyses

The sociodemographic variables statistically associated with the presence of anti-HBc after bivariate testing were the level of education (p = 0.04), provincial origin of women (p<0.001), their accommodation in dormitories (p<0.001), more than one sexual partner (p = 0.04), use of condoms and a low income (p = 0.02). Otherwise, the prevalence of anti-HBc was not significantly related to the level of knowledge about hepatitis B or with potential risk factors (Table 2).

**Table 2:** Association between socio-demographic characteristics, knowledge and risk factors with Anti-HBc. Ref = reference.

Variable		Anti-HBc Positive/Total (%)	Bivariate analysis			Multivariate analysis		
			OR	95% CI	P-value*	OR	95% CI	P-value
**Highest level of Education**	Illiterate/Primary	80/158 (50.63)	Ref	Ref		Ref	Ref	
	Secondary study	100/216 (46.29)	0.84	[0.55–1.26]	0.4	0.81	[0.52–1.26]	0.3
	Higher education	7/25 (28)	0.37	[0.15–0.95]	0.04	0.64	[0.21–1.91]	0.4
**Geographic origin**	Vientiane Capital	26/90 (28.88)	Ref	Ref		Ref	Ref	
	Provincial	161/309 (52.10)	2.67	[1.61–4.44]	<0.001	1.50	[0.81–2.78]	0.1
**Marital status**	Single	117/233 (50.21)	Ref	Ref		Ref	Ref	
	Married	60/146 (41.09)	0.69	[0.45–1.05]	0.08	0.67	[0.27–1.65]	0.3
	Divorced	10/20 (50.00)	0.99	[0.39–2.47]	0.9	0.47	[0.14–1.53]	0.2
**Have boyfriend**	No	72/147 (48.97)	Ref	Ref				
	Yes	115/252 (45.63)	0.87	[0.58–1.31]	0.5			
**Accommodation**	House	50/150 (33.33)	Ref	Ref	<0.001	Ref	Ref	
	Dormitory	118/205 (57.56)	2.71	[1.75–4.20]	<0.001	3.02	[1.53–5.95]	0.001
	Rent a room	19/44 (43.18)	1.52	[0.76–3.01]	0.2	1.29	[0.62–2.68]	0.4
**History of pregnancy**	No child, no abortion	118/254 (46.45)	Ref	Ref		Ref	Ref	
	Child, No abortion	24/54 (44.44)	092	[0.51–1.66]	0.7	2.34	[0.95–5.74]	0.06
	Abortion	45/91 (49.45)	1.12	[0.69–1.82]	0.6	2.55	[1.15–5.68]	0.02
Have heard of hepatitis*	No	129/273 (47.25)	Ref	Ref				
	Yes	58/126 (46.03)	0.95	[0.62–1.45]	0.8			
**Know the symptoms**	Do not know/poor knowledge	176/373 (47.18)	Ref	Ref				
	At least one	11/26 (42.30)	0.82	[0.36–1.83]	0.6			
**Know that Hepatitis B is curable**	No	178/378 (47.08)	Ref	Ref				
	Yes	9/21 (42.85)	0.84	[0.34–2.04]	0.7			
**Know Hepatitis is a severe disease**	No	178/376 (47.34)	Ref	Ref				
	Yes	9/23 (39.13)	0.71	[0.30–1.69]	0.4			
**Know someone infected in the family**	No	153/329 (46.50)	Ref	Ref				
	Yes	34/70 (48.57)	1.08	[0.64–1.82]	0.7			
**Contact with human blood**	No	151/325 (46.46)	Ref	Ref				
	Yes	36/74 (48.64)	1.09	[0.65–1.80]	0.7			
**Ever had Sexual intercourse**	No	57/131 (43.51)	Ref	Ref				
	Yes	130/268 (48.50)	1.22	[0.80–1.86]	0.3			
**Number of partners**	No sex	57/131 (43.51)	Ref	Ref		Ref	Ref	
	One	96/211 (45.49)	1.08	[0.69–1.68]	0.7	1.39	[0.78–2.50]	0.2
	More than one	34/57 (59.64)	1.91	[1.02–3.61]	0.04	2.12	[1.02–4.40]	0.04
**Use condom**	No sex	57/131 (43.51)	Ref	Ref				
	Always	25/38 (65.78)	2.49	[1.17–5.30]	0.01			
	Sometime	35/84 (41.66)	0.92	[0.53–1.61]	0.7			
	Never	70/146 (47.94)	1.19	[0.74–1.92]	0.4			
**Have STIs**	No	176/373 (47.18)	Ref	Ref				
	Yes	11/26 (42.30)	0.82	[0.36–1.83]	0.6			
**Income**	<1,700,000	167/342(48.83)	Ref	Ref		Ref	Ref	
	>1,700,000	20/57 (35.08)	0.56	[0.31–1.01]	0.05	0.69	[0.35–1.33]	0.2
**Age**	15–29	128/265 (48.30)	1.18	[0.78–1.80]	0.4			
	30–44	50/115 (43.47)	0.82	[0.53–1.27]	0.3			
	>45	9/19 (47.36)	0.96	[0.37–2.44]	0.9			

*Those variables with p≤0.2 were considered for inclusion into the multivariate analyses. “History of pregnancy” was considered an important variable and therefore was included in multivariate analyses despite not reaching p≤0.2 in bivariate analysis. “Using a condom” was correlated with “Number of partners” and therefore was excluded from the multivariate analysis

### Multivariate analyses

In the final model of multivariate analysis, three factors emerged as being significantly associated with the presence of anti-HBc: living in a dormitory, having more than one sexual partner, and a history of abortion (Table 2). Due to the low number of anti-HCV positive participants, no risk factor analyses were performed on these data.

## Discussion

This study aimed to investigate the prevalence of HBV and HCV infections among women working in garment factories in Vientiane, as well as the associated risk factors. The survey targeted this population on the assumption that it mostly consists of young women who live far from the protective environment of their families and who share a number of characteristics such as rural origin, low education level and socioeconomic status, which may make them more vulnerable to blood borne and/or sexually transmitted agents. A study in China showed that rural-to-urban migrant workers experience difficulties in adapting to their new environment and the socioeconomic stress [8].

Several features highlighted in the study population confirmed this vulnerability of our cohort: (i) half of the participants were housed in dormitories of the factory, (ii) their level of education including their knowledge in preventive health was low, (iii) their wages were low (about 1.2 million LAK or US $ 150 per month) [6][9]. Furthermore, all of these women were born well before 2001, when routine hepatitis B vaccination was introduced in the Expanded Program on Immunization of Lao PDR. All these factors foreshadowed a higher seroprevalence of markers of infection with HBV and/or HCV in these female workers than in the general population.

Regarding the seroprevalence of HBsAg, the rate of 4.0% is unexpectedly low. Although very close to the rate found among factory workers in Pakistan [10], it seems much lower than the expected prevalence in Lao PDR. Indeed, most serological surveys conducted so far highlighted rates of HBsAg carriage higher or equal to 8%; 8.7% among blood donors (2003–2005) [2], 8.2% among pregnant women from Luang Prabang and Vientiane in 2011 [3]and 8% among women attending antenatal clinics in Vientiane in 2013 [11]. The only discordant result was a 2.9% prevalence rate found in a nationwide survey conducted in 2012 on a representative sample of 965 mothers aged 15–45 years among which 71% were farmers [12], albeit tested by a point of care (rapid) test. As in our study, most women were of rural origin (78%), this discrepancy could reveal some inequality in the risk of exposure to HBV infection between the urban and rural components of the Laotian population. In rural areas, population densities are lower and the risk practices, such as multiple sexual partners or behaviors involving exposure to blood contamination, may be lower than in cities. Similar differences have been reported in Gabon [13] with a higher prevalence of chronic HBV in urban than in rural areas.

Interestingly, almost half of the participants (46.9%) are anti-HBc positive, which is close to the rates found in Lao PDR among women of similar age (49.5%) [3] or in blood donors (43.7%) [7]. HBV infections acquired later in life, after childhood, are generally cleared without progression to chronic disease and cirrhosis [1][14]. Hence the high anti-HBc positivity paired with a low HBsAg prevalence may reflect late HBV exposure after migration from the countryside to the city. This is consistent with our observation that anti-HBc positivity was significantly associated with risk factors for sexual transmission (multiple sex partners, history of abortion) and with dormitory accommodation. It is unlikely that sexual transmission occurs in the dormitories, but such an accommodation may promote risk behaviors such as spending nights outside with casual partners [9].

Unlike the carriage of HBsAg, the seroprevalence of anti-HCV antibodies (1.75%) was close to the previous estimate of 1.1% in Lao blood donors [2]. A survey of factory workers in Pakistan found a higher prevalence of HCV (6.35%) than HBV (1.98%) infection [10]. The main risk factors in Pakistan are the number of injections received and the reuse of syringes, poor knowledge of diseases lack of awareness of preventive measures, unsafe health care and promiscuity [15]. The low rate in Lao factory workers is consistent with the fact that risk factors for blood transmission (history of blood transfusions, tattoos, needles reuse and accidental blood exposures) were rare or absent in our study population, whilst injecting drug use is not common in Lao PDR. Furthermore, previous studies have shown that HCV prevalence may differ between males and females. Therefore it is difficult to directly compare our data with the general population [16][17].

Overall, the fear of discovering high rates of chronic HBV and / or HCV infection in this representative sample of Lao female workers has been belied by our findings. However, this does not rule out the reality of exposure to HBV infection for this vulnerable population.

Given this fact, several preventive measures could be implemented. First, routine vaccination against hepatitis B should be implemented upon hiring by the occupational health services, since at least 50% of these women may be susceptible to HBV infection [18]. Second, although it does not appear significantly related to the prevalence of anti-HBc, the low level of knowledge in this female working population should also be addressed by the preventive interventions. Educational programs for health must focus in particular on the prevention of STIs and the danger of high-risk practices of blood exposure [19]. Third, regarding the lifestyle of women workers, low-cost interventions could reduce the risks linked to their accommodation by making dormitories more attractive and user-friendly, like "at home", and avoiding women to become exposed outside the factory. This is a topic for research in the field of public health clearly worth exploring [6].

More generally, this study highlights the need to develop occupational health in Lao PDR, especially for women workers in garment factories, with a focus on information and prevention in the field of communicable diseases and reproductive health.

## Supporting information

S1 Fig Initial concept framework to show the factors hypothesized to impact on HBV and HCV infection in factory workers. (DOCX)

S1 Table Features of the garment factory workers enrolled in the study. (DOCX)

## Abbreviations

AIC: Akaike’s information criterion
Anti-HBc: Anti-HB core antibody
Anti-HBs: Anti-HB surface antibody
Anti-HCV: Anti-hepatitis C virus antibody
CI: Confidence Interval
ELISA: Enzyme-linked immuno-absorbent assay
GCP: Good Clinical Practice
HBsAg: Hepatitis B surface antigen
HBV: Hepatitis B virus
HCV: Hepatitis C virus
LAK: Lao Kip
Lao PDR: Lao People’s Democratic Republic
LRT: Likelihood Ratio Test
OR: Odds Ratio
STIs: Sexually Transmitted Infections
