# Supplementary material for: Seroprevalence and risk factors of hepatitis B and C virus infections in female workers of Lao garment factories
Source: PLoS One. 2018 Jul 16;13(7):e0199919. doi: 10.1371/journal.pone.0199919 (PMC6047780; doi:10.1371/journal.pone.0199919)
Supplement: S1 Fig — (DOCX) [file pone.0199919.s001.docx]

**S1 Figure. Initial concept framework to show the factors hypothesized to impact on HBV and HCV infection in factory workers.**

**Infections with HBV or HCV**

**Risk factors**

- Unprotected sex

- STIs

- Re-used needles

- Blood exposure

- Non-medical injections (drug use)

**Practice**

**-** Re-use needles

- Vaccination against HBV

- Protection from blood products

- Safe sex

**Knowledge**

**-** Hepatitis

- Risk factors

- Existence of vaccine

- Prevention

**Socio-demographic**

- Age

- Geographic origin

- Education

- Income

- Family situation
