# Supplementary material for: Seroprevalence and risk factors of hepatitis B and C virus infections in female workers of Lao garment factories
Source: PLoS One. 2018 Jul 16;13(7):e0199919. doi: 10.1371/journal.pone.0199919 (PMC6047780; doi:10.1371/journal.pone.0199919)
Supplement: S1 Table — (DOCX) [file pone.0199919.s002.docx]

S1 Table. Features of the garment factory workers enrolled in the study

| **Variables** | | **n (%)** |
| --- | --- | --- |
| **Sociodemographics** | |  |
| Highest level of education | Illiterate/Primary | 159 (39.75) |
|  | Secondary study | 216 (54) |
|  | Higher education | 25 (6.25) |
| Geographic origin | Vientiane Capital | 90 (22.5) |
|  | Provincial | 310 (77.5) |
| Marital status | Single | 233 (58.25) |
|  | Married | 147 (36.75) |
|  | Divorced | 20 (5) |
| Have boyfriend | Yes | 253 (63.25) |
| Accommodation | House | 150 (37.5) |
|  | Dormitory | 205 (51.25) |
|  | Rent a room | 45 (11.25) |
| **Knowledge** |  |  |
| Have heard of hepatitis | Yes | 126 (31.5) |
| Know the symptoms of hepatitis | Do not know/poor knowledge | 374 (93.5) |
|  | At least one | 26 (6.5) |
| Good knowledge of HBV and HCV transmission | Yes | 11 (2.75) |
| Believe that Hepatitis B can be cured | Yes | 21 (5.25) |
| Think that hepatitis is a severe disease | Yes | 23 (5.75) |
| Know there is HBV vaccine | Yes | 25 (6.25) |
| Know someone infected in the family | Yes | 70 (17.5) |
| **Risk factors and practice** |  |  |
| History of pregnancy | No child, no abortion | 255 (63.75) |
|  | Child, No abortion | 54 (13.5) |
|  | Abortion | 91 (22.75) |
| Using needles for non-medical purposes | Yes | 13 (3.25) |
| Accidental sharp injury | Yes | 3 (0.75) |
| Re-use needles | Yes | 4 (1.00) |
| Contact with human blood | Yes | 75 (18.75) |
| Number of partners | No sex | 131 (32.75) |
|  | One | 212 (53) |
|  | More than one | 57 (14.25) |
| Sex for money | Yes | 6 (1.5) |
| Tattoos | Yes | 9 (2.25) |
| Use condom | No sex | 131 (32.75) |
|  | Always | 38 (9.5) |
|  | Sometimes | 85 (21.25) |
|  | Never | 146 (36.5) |
| STIs | Yes | 27 (6.75) |
| Have been tested for hepatitis | Yes | 27 (6.75) |
| Have been vaccinated for HBV | Yes | 1 (0.25) |
| Would inform family if infected with hepatitis virus | Yes | 386 (96.5) |
| Would help infected family member | Yes | 389 (97.25) |
